# It takes a village: Community science informs tick encounter, pathogen, and exposure risk in North Carolina, USA

**DOI:** 10.1371/journal.pone.0352204

**Published:** 2026-07-24

**Authors:** Dayvion R. Adams, Kane Lawhorn, Allison N. Yackley, Kaylin S. Lewandowski, Martha O. Burford Reiskind, Alexis M. Barbarin, Michael H. Reiskind

**Affiliations:** 1 Department of Entomology and Plant Pathology, North Carolina State University, Raleigh, North Carolina, United States of America; 2 Genetic Engineering and Society Center, North Carolina State University, Raleigh, North Carolina, United States of America; 3 Department of Biological Sciences, North Carolina State University, Raleigh, North Carolina, United States of America; 4 Communicable Disease Branch, Division of Public Health, North Carolina Department of Health and Human Services, Raleigh, North Carolina, United States of America; University of Kentucky College of Medicine, UNITED STATES OF AMERICA

## Abstract

As tick-borne disease cases continue to increase over time, there is a growing need to understand the ecological and epidemiological factors that contribute to disease risk. North Carolina is currently experiencing a rise in cases, yet tick-borne research in the state remains limited. In this study, we developed a framework for a community science program in collaboration with 22 county public health agencies to recruit participants to submit incidentally encountered ticks. As part of kit submissions, participants also completed a form describing where and when they encountered the tick, as well as the behaviors that led to the encounters. Submitted ticks were tested for several putative bacterial pathogens, including *Borrelia burgdorferi*, *Ehrlichia* spp., and *Rickettsia amblyommatis*. We additionally assessed how advertising methodology and frequency by county health agencies influenced tick kit submissions and evaluated correlations between agency perceptions of project performance and submission rates. Over two years, we received 444 ticks across 319 unique submissions. While species distributions were largely consistent with prior observations, we report the first published instance of *Amblyomma americanum* within the mountainous region of NC, indicating a potential range expansion into cooler, higher-altitude areas. Relative risk modeling identified recreational activities as consistently associated with higher likelihood of tick kit submissions. Multiple advertising types appeared to influence the number of kits submitted; however, these results should be interpreted cautiously due to small sample sizes. Overall, our study demonstrates a successful collaboration with state and county health agencies to engage the community in tick-borne disease research. Future studies should build on this framework to further optimize participation.

## Introduction

Tick-borne disease (TBD) cases are rising in the United States due to changes in climate, landscape, and human behavior [1]. Lyme disease, the most commonly reported tick-borne disease, continues to increase in incidence, including in North Carolina, where the range of *Borrelia burgdorferi* is expanding southward [2]. Incidence of other tick-associated diseases, including ehrlichiosis and spotted fever group rickettsiosis, is also increasing [1]. While climate likely influences tick range expansions and vector proliferation [3], human behavior further shapes pathogen transmission [4,5]. Rapid population growth across North Carolina and the southeastern United States drives urban and suburban development in a region with high deer densities [6–8], bringing people into closer contact with deer, a key tick host, and pathogen-carrying ticks. The state’s abundant natural landscapes make outdoor recreation a frequent pastime for residents and visitors alike, increasing opportunities for tick exposure [9–11]. Together, these factors help explain the notable rise in TBDs observed in North Carolina in recent years. Despite this, current knowledge of tick distributions, pathogen prevalence, and human exposure risk in North Carolina remains limited, and only a handful of studies in the past few years have begun to address these gaps [2,10].

Community science, previously referred to as citizen science, represents a valuable data-gathering approach for TBD studies that rely on passive surveillance to address large-scale ecological and public health questions. To date, this approach has been applied across nearly all major vector-borne disease systems [12–14]. For example, community-based collection of triatomine bugs in the southern United States has substantially improved understanding of species diversity and Chagas disease transmission risk in Texas [15]. Similarly, community science approaches have also been used to assess *Ixodes scapularis* (Say) encounter rates and Lyme disease risk in high incidence regions, such as with the “Tick App” project [16]. Although community science studies can be logistically complex and resource-intensive depending on study design, they provide unique advantages by generating large spatial datasets, improving understanding of ecological disease risk, and engaging the public through education and participating in scientific research.

To date, no studies have comprehensively evaluated tick distributions across North Carolina’s diverse landscapes, as most efforts have focused on subsets of counties within individual ecoregions [17,18]. Other studies have restricted sampling or reporting to a single tick species [2]. Consequently, much of the existing knowledge relies on passive surveillance, such as tick collections from deer or submissions to local and state agencies, which allows for broader geographic coverage [19–21]. While these approaches provide valuable insights into localized patterns and facilitate species distribution modeling, they offer an incomplete picture of statewide tick diversity and tick-borne disease risk and can quickly become outdated as tick and pathogen ranges shift in response to environmental change [2,22].

Only a subset of tick species in North Carolina are sufficiently abundant to pose human and animal health risks. *Ixodes scapularis*, the vector of Lyme disease, occurs statewide, although sustained *B. burgdorferi* transmission appears largely restricted to the mountain and Coastal Plains ecoregions [23]. In contrast, *Amblyomma americanum* (Linnaeus) is likely the most abundant tick in the state, with a range extending from the Piedmont through the Coastal Plains [10,24], and is associated with multiple human pathogens, including *Ehrlichia chaffeensis* [25], *Ehrlichia ewingii* [26], Bourbon virus [27], putative spotted fever group rickettsiosis agent *Rickettsia amblyommatis* [28], and as the cause of alpha-gal syndrome [29]. *Dermacentor variabilis* (Say) is also common throughout the state [10,30], and although it has been historically linked to Rocky Mountain spotted fever (*Rickettsia rickettsii*), this pathogen is now rarely detected in field-collected ticks [31], raising concerns about misdiagnosis of other spotted fever rickettsioses or other tick species as potential vectors [32]. Less frequently encountered species still warrant attention, including *Amblyomma maculatum* (Koch), a vector of *Rickettsia parkeri* rickettsiosis in the Piedmont and Coastal Plains [33], and the invasive *Haemaphysalis longicornis* (Neumann), which has recently expanded into North Carolina [34–36]. Although *H. longicornis* human bites are rarely reported in the United States [37,38], it is a known vector of severe fever with thrombocytopenia syndrome virus in its native range [39,40].

In this study, we partnered with the North Carolina Department of Health and Human Services Communicable Disease Branch and multiple county health departments to solicit tick submissions and investigate the ecology of tick-borne diseases in North Carolina. Our primary objectives were to characterize the geographic distribution of ticks and their associated pathogens across the state’s three major ecoregions, identify human behaviors strongly associated with tick encounters, and evaluate both the perceived and observed effectiveness of the project as a community science engagement tool. We employed a flexible study design that enabled the collection of physical tick specimens while minimizing barriers to public participation. As the first study of its kind conducted statewide in North Carolina, this work provides updated context on tick-borne disease risk and transmission, as well as insight into human behaviors that contribute to tick encounters. Collectively, these findings contribute to the growing body of literature in the southeastern United States aimed at reducing tick exposure and promoting public health across the state.

## Materials and methods

### Community science project

**Fig 1:**
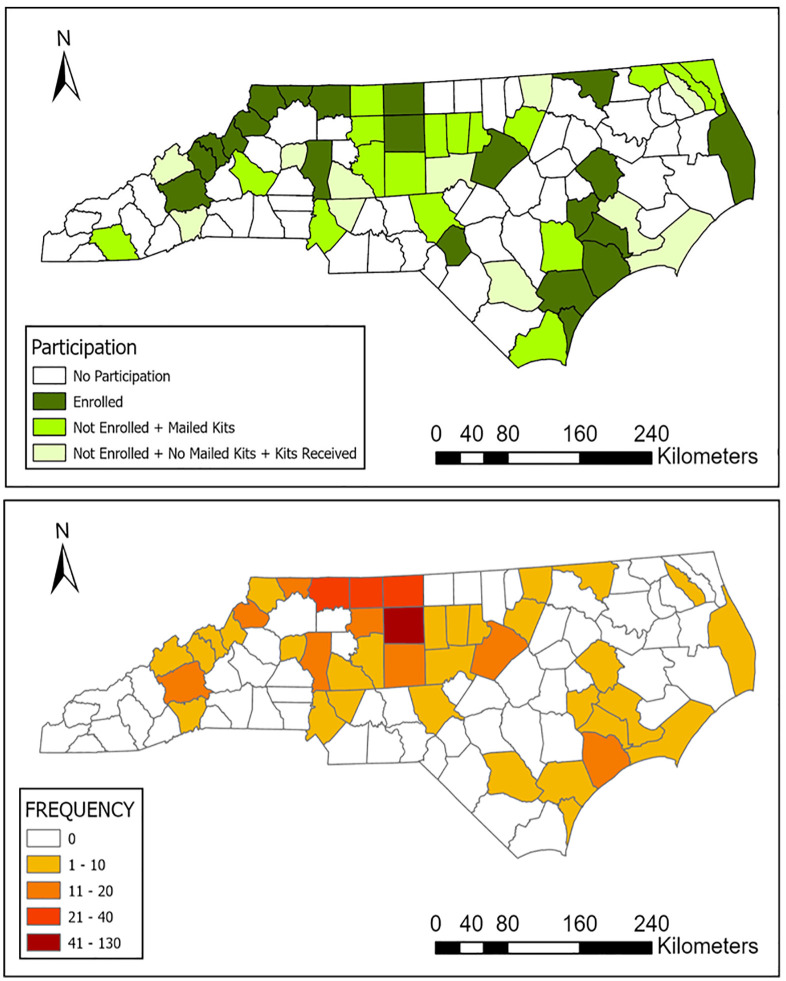
Choropleth maps showing county-level participation categories (A) and the frequency of tick kit submissions (B). Counties were classified as: (1) no participation; (2) enrolled in the project with requests for mailed kits; (3) not enrolled but with requests for mailed kits; or (4) not enrolled and without mailed kit requests but with kit submissions returned to NCSU. In total, 46 (46%) of counties returned kits. Basemap source: NC OneMap.

As part of a collaboration with the North Carolina Department of Health and Human Services (NCDHHS) and several county health departments (hereafter referred to as counties or county health agencies), we established a contributory community science project to solicit tick submissions from naturally occurring encounters by residents (hereafter referred to as residents or participants). Participating counties were recruited through multiple avenues, including direct email, word of mouth, and requests following a project presentation at an NCDHHS communicable diseases event. County enrollment in our project was voluntary and 22 participating North Carolina counties out of 100 included Alleghany, Ashe, Avery, Brunswick, Buncombe, Dare, Guilford, Hoke, Iredell, Jones, Lenoir, Mitchell, New Hanover, Northampton, Onslow, Pender, Pitt, Rockingham, Surry, Wake, Watauga, and Yancey (Fig 1). Tick submissions from the public were accepted over a two-year period, from November 1, 2023, through October 31, 2025. County health departments served as kiosk locations, with one site per county where residents could pick up “tick kits” and later use them to submit ticks to North Carolina State University (NCSU). County health department staff also addressed community questions about the project and coordinated with NCSU to restock kiosks as needed. We provided Spanish-language materials to one county upon request due to its large Spanish-speaking population. To participate, counties were required to place the tick kit kiosk in a visible, easily accessible location within the health department offices and advertise the project at least once, without specific requirements for how the project was advertised.

**Fig 2:**
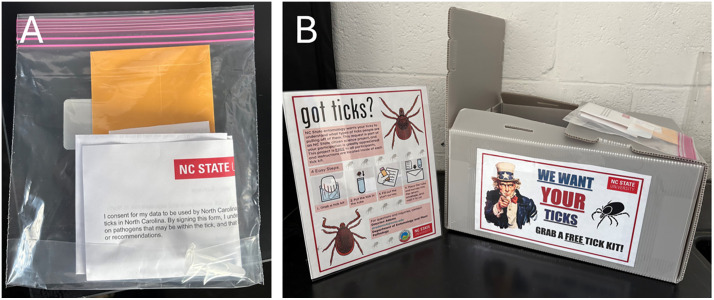
Tick kits (A) distributed to study participants. Each kit contained a 1.5 mL microcentrifuge tube filled with 70% ethanol, a prepaid return envelope, a tick encounter survey, and an informed consent form. Kits were made available through “kiosks” (B) placed at participating county health agencies.

We designed tick kits to provide a quick and simple way for community participants to participate in the contributory project. Each kit included one or two 1.5 mL microcentrifuge tubes filled with 70% ethanol, a tick collection survey, a research consent form, participation instructions, and a coin envelope with prepaid postage and the NCSU mailing address. We placed all materials inside a resealable sandwich bag (Fig 2). If residents were unable to easily access a participating county health department kiosk, they could contact our research team directly to request kits by mail. We specified in the instructions that we only accepted ticks collected directly from humans, not from pets. Residents could submit ticks that were attached (embedded) or crawling on them at the time of collection. Ticks could be submitted on behalf of another individual (e.g., a child or spouse), provided that the survey responses reflected the person from whom the tick was collected. We advised residents not to seek out ticks intentionally but to report those encountered incidentally. The accompanying survey, hereafter referred to as tick survey, asked residents about their tick knowledge, collection details (for example, when and where), human behavior, and general demographic information (S3 File). We immediately decoupled submissions from their survey responses, and we did not collect any personally identifiable information from participants. We also developed and distributed a post-participation survey to the participating county health departments, hereafter referred to as the provider survey (S4 File). For provider surveys, we requested that all individuals involved in the project and kit distribution process complete the survey, and in some counties, this included multiple respondents. This survey collected information on advertising type and frequency, motivations for enrollment and participation, and perceptions about community engagement, participation, and project effectiveness. Both surveys and all data collection and handling methodologies were approved by the North Carolina State University Institutional Review Board (protocol #25948).

### Tick processing and molecular diagnostics

Upon receiving each tick kit by mail, we identified ticks to species using established morphological keys [41–46]. We then stored ticks at −20°C in 70% ethanol until DNA extraction. We bisected all adult tick specimens and reserved one half in case of extraction failure, while nymphal ticks were extracted whole. Because few ticks were submitted per individual submission, we extracted DNA from each tick individually rather than pooling specimens. To account for potential DNA degradation during mail transit, we quantified all DNA extracts and excluded samples (n = 2) with DNA concentrations below 0.01 ng/mL from molecular analyses.

We conducted species-specific real-time PCR (qPCR) assays on all submitted ticks, except for *Ixodes* spp., which we sent directly to the Centers for Disease Control and Prevention (CDC) for pathogen screening of *B. burgdorferi* sensu stricto, *Borrelia miyamotoi*, *Borrelia mayonii*, *Babesia microti*, *Ehrlichia muris eauclairensis*, and *Anaplasma phagocytophilum* due to a statewide *Ixodes scapularis* surveillance agreement. We screened *A. americanum* for *R. rickettsii*, *R. amblyommatis*, *E. chaffeensis*, and *E.ewingii*; *D. variabilis* for *R. rickettsii*; and *A. maculatum* for *R. parkeri*. Our qPCR methods followed those described in Adams et al. (2025)(18), except for the *R. parkeri* assay, which we performed using the Rpa129F and Rpa224R primers and the Rpa188 FAM (6-carboxyfluorescein) probe [47].

### Survey data extraction

Because the tick survey questions were open-ended, we received a wide range of responses. We grouped and coded these responses into categories for analysis to identify the most prevalent participant behaviors. We recorded both perceived and actual tick species to assess participant prior knowledge. We recorded both the county of collection and county of residence to assess whether participants encountered ticks while traveling. Behavioral categories coded from survey responses included working outdoors (e.g., yard work); a broad outdoor recreation category encompassing multiple forms of outdoor activity; tick encounters at private residences or properties; presence of pets or other animals (e.g., dog walking); gardening; hunting or fishing; a combined category of hiking, walking, or camping; walking through or playing in residential yards; and other less common or unspecified recreational activities (e.g., mountain biking, sports)(S1 Table in S2 File). In our survey, we asked participants to report the habitat where the tick was collected, if known, and coded responses such as indoors, forested trail, forest edge, grass field, grass field edge, residential yard, buildings, and near a body of water (S1 Table in S2 File). We collected demographic information, including age, gender, and ethnicity to characterize participant backgrounds. We also recorded collection time and date when provided. If no collection date was provided, we used the signed and dated consent form as a proxy for collection date. The final survey question asked whether participants wanted us to follow up with general information about the species. However, we clarified on the survey that we could not provide tick-pathogen results as NCSU cannot influence participants’ medical decision-making. All tick surveys were reviewed by at least two researchers to ensure consistency and quality.

Our provider surveys included a mix of open-ended questions, Likert-scale items, and multiple- response questions (select all that apply) (S4 File). We recorded the responder’s county and linked it to the number of submissions received from that county. Open-ended responses regarding advertising types were coded into the following categories: no advertising, social media (e.g., Facebook), community health department website posts, press releases, community outreach, email or email newsletters, word of mouth, and dedicated signage (beyond what was provided). We provided multiple options for potential motivations for participation, along with an open-ended choice, to understand why counties chose to enroll in our project (S4 File, Question 9). These responses were coded into the following categories: concern about TBDs spreading into their communities, concern about TBDs already present, desire to contribute to science, interest in raising public awareness of TBDs, and interest in participating in a citizen/community science project. Several survey questions used a Likert-scale format, including participants’ concern about TBDs in their communities, frequency of TBD reports from residents, perceived community interest in the project, and perceived effectiveness of the project in engaging the community on TBDs. Likert responses were then assigned numerical values from 0 to 4 according to participants’ selections.

### Statistical and spatial analyses

To determine what coded behaviors and habitat types were associated with higher tick submission counts, we used a generalized linear mixed modeling (GLMM) approach to conduct a relative risk analysis. We assessed potential collinearity among variables and removed any with a correlation coefficient greater than 0.60. Our data were best modeled using a negative binomial distribution with a log link function, as absence data were not available with our submission design. We included an offset term for county population to account for variation in population sizes among participating counties. Given the large number of potential predictor variables, we identified the best-fitting variables using a stepwise approach based on Akaike’s Information Criterion (AIC) and likelihood ratio test elimination. A list of all recorded and considered predictor variables is provided in S1 Table in S2 File. To compare the effects of variables within the model, we calculated incidence rate ratios (IRRs), which quantify the rate of change associated with each variable while holding all other variables constant. To test whether any motivation type was represented significantly more than others in our provider survey, we used a Chi-square goodness-of-fit test. We used GLMM analysis to evaluate which local health department advertising methods most strongly contributed to tick submissions. Models employed a negative binomial distribution with a county-level population offset and advertisement frequency to assess potential interactions between advertising method and frequency. We also tested whether advertising frequency correlated with the number of submissions per county using Kendall’s tau. We utilized Spearman’s rho to assess whether county health department Likert-scale perceptions (described above) correlated with the number of submissions received. We processed and analyzed all data using R statistical computing software v4.5.2 (R Foundation for Statistical Computing, Vienna, Austria). We used an alpha value of 0.05 for judging statistical significance. We calculated correlations using the base stats package and performed GLMMs using the glmmTMB package [48]. We created figures using ArcGIS Pro (Esri, Redlands, CA, USA; version 3.4) and the ggplot2 package in R [49].

## Results

### Tick kit submissions

**Fig 3:**
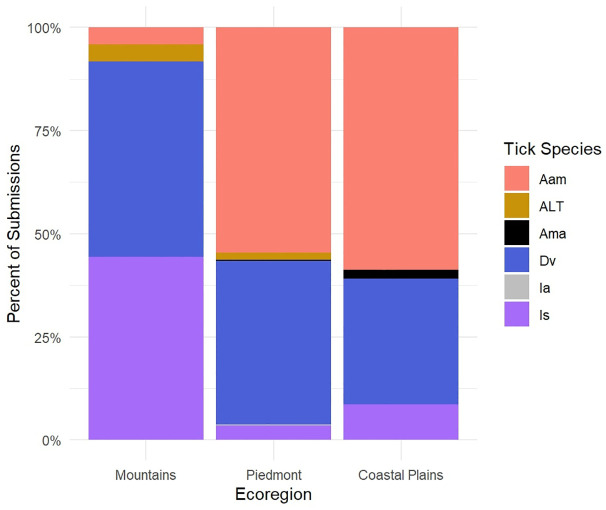
Stacked bar chart showing the percentage of tick species submissions by ecoregion. Species abbreviations in the legend are as follows: Aam, *A. americanum* (lone star tick); ALT, *H. longicornis* (Asian longhorned tick); Ama, *A. maculatum* (Gulf Coast tick); Dv, *D. variabilis* (American dog tick); Ia, *I. affinis*; and Is, *I. scapularis* (blacklegged tick).

**Fig 4:**
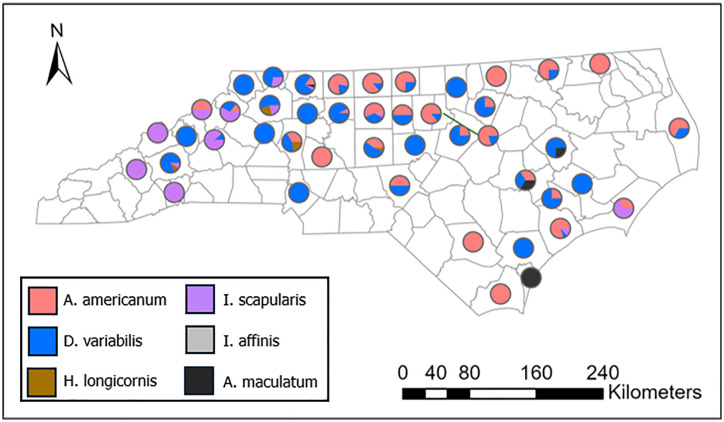
County-level breakdown of the relative proportion of tick species submitted by participants. Submission counts varied by county, with some counties represented by a single tick. Basemap source: NC OneMap.

Our community science efforts yielded 323 unique submissions from 46 counties across North Carolina (46% of all counties; Fig 1), including four out-of-state submissions (Virginia and Missouri), which were excluded from analyses. In total, we provided 3,094 tick kits to participating counties and received 319 submissions from North Carolina (10.3% return rate). A county-level breakdown of kits provided and received is presented in S2 Table in S2 File. These North Carolina submissions contributed 444 individual data entries. Three submissions consisted of non-tick arthropods, four submissions were too damaged for tick species identification, and 35 submissions were missing tubes upon arrival due to damaged postal packaging. We identified a total of 405 ticks comprised of 187 *A. americanum* (61 females, 73 males, 44 nymphs, and 9 larvae), 161 *D. variabilis* (89 females, 72 males), 46 *I. scapularis* (33 females, 10 males, and 3 nymphs), 8 *H. longicornis* (5 females, 3 nymphs), 2 adult *A. maculatum* (1 male, 1 female), and 1 female *Ixodes affinis* (Neumann). We received 286 ticks from the Piedmont ecoregion, 72 from the Mountains, 46 from the Coastal Plains, and one submission from an unknown county of collection (Fig 3). A county-level map showing the relative proportions of each species submitted is presented in Fig 4, with species-specific distribution maps provided in S1–S5 Figs in S1 File. Thirty-nine ticks were partially or fully engorged. Most submissions contained a single tick; however, some participants submitted multiple ticks, with a maximum of nine ticks in a single submission.

### Molecular screening results

**Fig 5:**
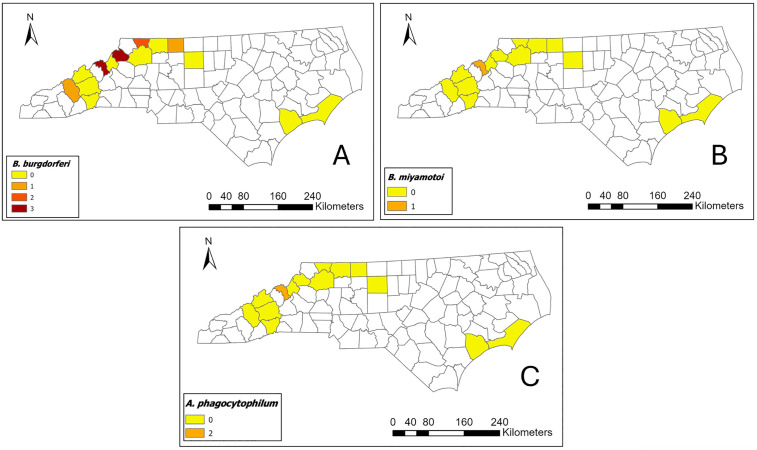
Choropleth maps showing county-level *I. scapularis* submissions that tested positive for *B. burgdorferi* (A), *B. miyamotoi* (B), and *A. phagocytophilum* (C). Basemap source: NC OneMap.

**Fig 6:**
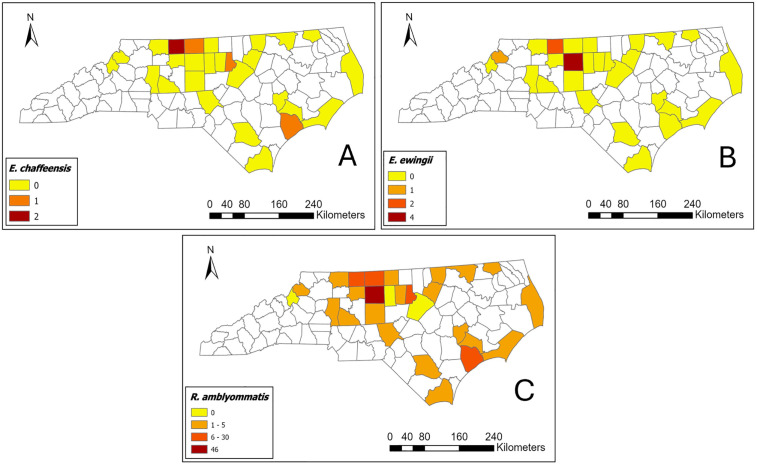
Choropleth maps showing county-level *A. americanum* submissions that tested positive for *E. chaffeensis* (A) and *E. ewingii* (B), and *R. amblyommatis* (C). Basemap source: NC OneMap.

In total, we screened 183 *A. americanum* submissions (137 adults and 46 nymphs), and the CDC screened 47 *I. scapularis* submissions. Among *A. americanum*, 6 samples (3.22%; 5 adults, 1 nymph) tested positive for *E. chaffeensis*, 7 (3.78%; 4 adults, 3 nymphs) for *E. ewingii*, and 146 (78.49%; 115 adults, 31 nymphs) for *R. amblyommatis*. Among *I. scapularis*, 10 samples (21.28%; 8 adults, 2 nymphs) tested positive for *B. burgdorferi*, 2 (4.26%; 2 adults) for *A. phagocytophilum*, and 1 (2.13%; 1 adult) for *B. miyamotoi*, while all samples tested negative for *B. mayonii*, *B. microti*, and *E. muris eauclairensis*. All *D. variabilis* were negative for *R. rickettsii.* One (50%) *A. maculatum* was positive for *R. parkeri*. Pathogen prevalence varied geographically, and county-level distribution patterns are presented in Figs 5 and 6, highlighting areas with higher proportions of infected ticks and spatial trends in exposure risk.

### Tick submission survey summary results

Participant tick survey responses varied widely in knowledge, detail, and attitude, and participant demographics spanned a range of ages, genders, and ethnicities. Females accounted for 186 submissions (56.5%), males for 140 (42.6%), non-binary participants for 2 (0.6%), and 1 participant (0.3%) did not report their gender. White participants accounted for 298 submissions (92.3%), Black participants for 5 (1.5%), Hispanic or Latino participants for 3 (0.9%), Asian participants for 1 (0.3%), and mixed-ethnicity participants for 2 (0.6%), and 14 participants (4.3%) did not report their ethnicity. Ticks were collected from participants with ages ranging from 3 to 90 years, with a median age of 58 years (IQR: 41–68); two submissions did not report age, and the highest representation occurred among individuals aged 55–60 years (S6 Fig in S1 File). Participants requested follow-up information 167 times (51.7%), while 156 participants (48.3%) declined follow-up. Of the ticks we could identify (i.e., not damaged or missing), participants correctly identified 178 ticks (43.0%) and incorrectly identified 236 ticks (57.0%). Participants provided collection dates for 164 ticks (40.5%), we estimated dates using consent forms for 211 ticks (52.1%), and no date was provided or could be estimated for 30 ticks (7.4%). Participants provided collection times for 378 ticks (93.3%), and no collection time was provided for 27 ticks (6.7%).

Ticks were submitted throughout the year, but submission phenology varied by species. Only the three most common species were submitted in sufficient numbers to characterize species-specific phenology and collection activity. Submissions of *A. americanum* occurred from March through September, with peak activity from April to June, whereas *D. variabilis* exhibited a similar seasonal pattern but peaked slightly later, from May to July (S7 Fig in S1 File). In contrast, *I. scapularis* submissions exhibited a multi-peak pattern, with peaks in cooler months (October to April) corresponding to adult host-seeking activity and a smaller summer peak corresponding to nymphal activity (S7 Fig in S1 File). Collection times for all three species were generally similar, spanning from 08:00–19:00. However, *A. americanum* and *D. variabilis* peaked during the warmest parts of the day, between 12:00 and 18:00, whereas *I. scapularis* peaked earlier, between 09:00 and 12:00 (S8 Fig in S1 File).

### Tick submission survey modeling results

Our modeling of tick submissions identified a broad range of behaviors and habitat types as significant predictors of tick submissions and relative risk (Table 1). Because outdoor recreation was significantly correlated with several recreational subcategories, we excluded this broader category from the final models. The best-fit negative binomial model included the following predictors, listed in order from largest effect size to smallest effect size: hunting and fishing, other recreational activities, ticks found indoors, grass field edges, forest edges, residential yards, forest trails, hiking/walking/camping (treated as a single category), owning animals, walking or playing in residential yards, grass fields, and ticks collected from private residences or property. These predictors were recurrent themes among open-ended survey responses. Incidence rate ratios used for relative comparison are presented in S9 Fig in S1 File.

**Table 1:** Best‐fit negative binomial GLMM results for tick kit submissions, highlighting commonly reported behavioral and environmental risk factors. Predictors are ordered by the magnitude of their estimated coefficients. In addition to these predictor variables, we also included a county population offset variable to account for variation in population size among participating counties.

Predictor	Estimate	SE (±)	Z-val	P-val	IRR
Hunting/Fishing	1.200	0.352	3.407	<0.001	3.321
Other Rec	1.131	0.345	3.279	<0.001	3.101
Found Indoors	−0.805	0.259	−3.103	<0.001	0.447
Grass Field Edge	−0.596	0.207	−2.880	<0.001	0.551
Forest Edge	−0.421	0.144	−2.929	<0.001	0.656
Yard	−0.420	0.132	−3.170	<0.001	0.657
Forested Trail	−0.395	0.124	−3.176	<0.001	0.674
Hike/Walk/Camp	0.349	0.082	4.235	<0.001	1.418
Owns Animals	0.295	0.134	2.192	<0.001	1.343
Walking/Playing in Yard	0.267	0.126	2.124	<0.001	1.306
Grass Field	−0.245	0.089	−2.739	<0.001	0.783
Private Residence	0.213	0.099	2.153	<0.001	1.238

### Kit provider survey results

We received 17 responses from 16 of the 22 participating counties in our post-participation provider survey. Respondents reported learning about the project through four main channels: an email announcement from NCDHHS (47%), a presentation by our NCDHHS collaborator at a statewide communicable disease meeting (29%), word of mouth (24%), and resident-driven interest (6%). When asked about motivations for participating, respondents consistently selected all five categories, with public awareness of TBDs reported most often (88%), followed by a desire to contribute to science (82%), concern about TBDs already present in their counties (76%), concern about TBDs spreading into their counties (65%), and interest in participating in a citizen science project (59%). There were no significant differences in motivation selection (χ² goodness-of-fit test: χ² = 1.3651, df = 4, p = 0.8502). Advertising frequency varied by county, ranging from zero to more than five instances, and was not significantly correlated with the number of tick submissions received (Kendall’s τ = −0.164, p = 0.3964). Generalized linear mixed models assessing advertising method and frequency identified no significant predictors (Table 2). Similarly, respondents’ perceptions of project effectiveness (Spearman’s ρ = −0.404, p = 0.1708), perceived community interest (ρ = −0.465, p = 0.109), concern about TBDs (ρ = 0.274, p = 0.3652), and frequency of reported TBD cases (ρ = −0.103, p = 0.7358) were not significantly correlated with submission counts.

**Table 2:** Results of the GLMM assessing the effects of advertising type by local health agencies on the number of tick kit submissions received. Only counties that completed the follow-up provider survey were included. Predictors are ordered by the magnitude of their estimated coefficients. Reference levels for binary predictors are shown in parentheses, with “Y” indicating a “Yes” (coded as 1).

Predictor	Estimate	SE (±)	Z-val	P-val
Community Outreach (Y)	7.98	10.93	1.52	0.13
Press Release (Y)	6.77	7.79	1.66	0.10
None (Y)	5.80	8.26	1.24	0.22
Sign (Y)	3.04	3.69	0.92	0.36
Social Media (Y)	2.60	1.77	1.40	0.16
Website (Y)	1.08	1.05	0.08	0.94
Advertising Frequency	0.87	0.26	−0.48	0.48
Word of Mouth (Y)	0.68	0.45	−0.59	0.18
Newsletter/Email (Y)	0.45	0.52	−0.70	0.49

## Discussion

This study used a community-based, collaborative approach over a two-year period to characterize tick encounter patterns across North Carolina, a state experiencing rapid population growth [50,51]. Despite the relatively short study duration, this framework enabled broad spatial coverage and provided insight into statewide patterns of tick and pathogen distributions, human behaviors associated with tick exposure, and variation in participation among county health departments. These findings highlight the value of community science for generating timely, large-scale surveillance data that would be difficult to obtain through traditional field-based approaches alone. Collectively, our results support the use of similar models to inform the development of larger, sustainably funded tick-borne disease surveillance programs in regions experiencing high and increasing disease burden.

Tick distribution patterns from submissions generally reflected expected statewide trends. For example, *I. scapularis* was submitted from counties across all ecoregions but occurred most frequently in the mountains and along the Virginia border (Fig 4, S3 Fig in S1 File), consistent with previous studies [2,20]. In contrast, *D. variabilis* was submitted from across the state, with the highest submission densities in the mountainous ecoregion and fewer submissions from the Piedmont (Fig 4; S1 and S2 Figs in S1 File). This pattern aligns with previous collections from the Piedmont of North Carolina and Virginia, where *D. variabilis* occurs at far lower rates than *A. americanum* [19,52,53], though the factors driving its relatively low abundance remain unclear. Conversely, *A. americanum* was collected at high densities in the Piedmont and Coastal Plains regions (S1 Fig in S1 File), supporting its dominance in the state, and it was also submitted from the mountainous region, where it has not been previously reported to our knowledge [54]. These submissions suggest that *A. americanum* may be expanding into western North Carolina, a region characterized by higher elevation and cooler climatic conditions.

Our *B. burgdorferi* pathogen screening largely reflected prior findings in North Carolina. Although many *I. scapularis* were submitted from other ecoregions, *B. burgdorferi*–positive ticks were found only in the mountains, except for one sample near the Virginia border (Fig 5). This aligns with Garshong, Adams (2), who documented southward expansion of the this species along the Blue Ridge Mountains, likely representing the northern clade responsible for *B. burgdorferi* transmission in the northeastern and midwestern United States. Regional differences in transmission ecology may exist, as southern clade *I. scapularis* more often feed on lizards rather than *Peromyscus leucopus* (Rafinesque) [55], the primary reservoir of *B. burgdorferi*. Despite the absence of infected ticks in other ecoregions in our study*, B. burgdorferi*-infected ticks may still be collected in these areas, though at lower rates than western North Carolina [56].

Following the geographic pattern observed for *B. burgdorferi*, *Ehrlichia*-infected *A. americanum* also showed spatial distribution, occurring mainly in the Piedmont despite frequent *A. americanum* submissions from the Coastal Plains (Fig 6). A similar pattern of clustering of human ehrlichiosis cases in the Piedmont, but not the Coastal Plains, was reported by Brown Marusiak, Giandomenico [57]. Taken together, this may suggest that the parasite is not prolific in the coastal region despite the abundance of *A. americanum* in this ecoregion. Together, these results may suggest that the pathogen is less prevalent in the Coastal Plains despite the abundance of *A. americanum* in this ecoregion. However, our sample sizes were low and uneven across counties and regions, and these findings should therefore be interpreted with caution. In contrast, *R. amblyommatis*–infected *A. americanum* from our study ticks exhibited high prevalence across their widespread geographic range (Fig 6). While the pathogenicity of *R. amblyommatis* in humans remains unclear [28,58,59], it may contribute to the broader pattern of spotted fever rickettsiosis cases noted by Brown Marusiak, Giandomenico [57] and the ongoing increase in cases across the eastern United States. Very few studies have detected *R. rickettsii* in nature, suggesting it is relatively rare and unlikely to be driving the rise in human cases [31,60–62]. Additionally, *A. maculatum* is collected infrequently in North Carolina [10], but *R. parkeri*-infected ticks may still play a role in the overall increase in spotted fever rickettsiosis incidence [33].

The results of our tick survey model highlight the importance of incorporating human behavior and environmental data when evaluating tick encounter risk. Participants most often attributed tick encounters to recreational activities such as hunting and hiking, with four recreational behaviors retained in the final model and exhibiting relatively high IRRs (Table 1 and S9 Fig in S1 File), indicating strong associations with tick submissions. While these activities inherently increase exposure by placing individuals in tick habitats, our models assessed the relative risk of submissions, and reports of recreational behavior may therefore also reflect reporting bias. Individuals who frequently recreate outdoors may be more likely to perform tick checks and subsequently submit ticks. Similar patterns have been reported previously, with prior tick exposure strongly associated with frequent tick checks but more weakly associated with other protective measures such as repellent use [63]. Additionally, participants may modify their behavior to reduce exposure, including avoiding areas of high tick density [64] and adopting evidence-based behaviors such as staying on established recreational trails [10]. Together, these findings emphasize the combined roles of personal protective measures and behavioral modification, including avoidance behaviors, in reducing tick bite risk among people engaging in outdoor recreation within tick habitats.

In addition to recreational behaviors, participants frequently reported several habitat types that were included in the final model. Although many of these habitats are well recognized as suitable tick environments, including edge habitats, forests, and grass fields [52,65], all were less frequently represented among submissions, as indicated by IRRs < 1 (Table 1 and S9 Fig in S1 File). This pattern suggests that submission rates may be more strongly driven by participant behavior and reporting tendencies than by habitat-specific encounter risk alone. Nevertheless, a substantial number of submissions originated from private residences (63.06%, n = 280) and residential yards (38.96%, n = 173), indicating that routine, peridomestic exposure to ticks is common in suburban to rural areas of North Carolina. Similar residential exposure patterns have been documented in the northeastern and midwestern United States, where Lyme disease incidence is high [4,66], but remain poorly characterized in the southeastern United States. As population growth and human-wildlife interactions due to habitat fragmentation continue to increase in North Carolina [6,67], the frequency of peridomestic tick encounters will rise, underscoring the importance of targeted public health messaging.

The design of our study was distinctive in its collaboration with county health agencies, which have extensive reach within local communities. We intentionally designed the project without strict implementation guidelines, allowing participating agencies to exercise discretion in advertising and community engagement strategies. While none of the advertising methods used by county health agencies produced a statistically significant increase in participant recruitment, community outreach and press releases showed the largest estimated effects for increased participation (Table 2). In particular, a press release in Guilford County coincided with a substantial increase in submissions, requiring multiple kiosk refills and frequent direct communication with interested participants. The press release also reached neighboring counties and, following communication and coordination with North Carolina State University, generated resident-driven interest that ultimately led to Rockingham County’s enrollment. These observations suggest that when residents are informed about community science projects involving ticks, they are highly motivated to participate. Interestingly, residents were willing to move across geopolitical boundaries (e.g., rural-urban gradients, county boundaries) in order to participate. This observation is evidenced by the receipt of kits from eleven counties that were not directly enrolled in the project, highlighting the strength of this approach for engaging communities across North Carolina. Counterintuitively, counties that reported no active advertising also received high numbers of submissions, which may indicate underlying differences in public concern or awareness of tick-related health risks (Table 2). However, these results should be interpreted cautiously, as the model was based on a limited number of county health agency responses (n = 17). Future studies that actively control advertising methods and intensity will be necessary to more rigorously evaluate their effects on community participation.

Large-scale community science projects often depend on collaboration across multiple entities, particularly when research goals require broad geographic coverage and sustained public engagement. In this study, partnerships with county health and vector control agencies enabled effective recruitment across North Carolina, with participation from 22% of county health departments spanning multiple ecoregions (Fig 1), thereby allowing meaningful evaluation of regional variation in tick communities. Although no single county enrollment motivation was significantly more common than others, public awareness of tick-borne diseases and a desire to contribute to science emerged as the most frequently reported drivers of participation. Concern about tick-borne disease risk in an emerging-risk state is unsurprising, particularly given that many county health and vector control agencies already actively conduct outreach and education on tick-borne diseases [68], allowing research efforts to be integrated directly into existing community-facing public health initiatives. The strong interest in contributing to science further suggests a willingness among both residents and agencies to collaborate with researchers to address vector-borne disease risk. This is reinforced by the finding that all county health agency respondents in our study indicated interest in participating in future vector-borne disease projects. Collectively, these results highlight the value of partnerships with local public agencies, whose regional expertise and established community trust can help overcome surveillance and engagement barriers [69] that often limit academic-led field studies. Integrating community science with local public health infrastructure may therefore represent a scalable and effective model for addressing persistent surveillance gaps in regions experiencing increasing vector-borne disease risk.

While community science studies like ours can generate large-scale geographic and temporal data, they are not without limitations. In this study, ticks were collected by participants rather than researchers, and respondents were asked to recall where, when, and what they were doing when they encountered ticks, introducing potential recall bias [70]. We attempted to mitigate this by using alternative methods, such as estimating collection dates from informed consent forms when participants did not provide them, but future studies could further improve accuracy through real-time digital reporting or GPS-based logging of tick encounters. Another limitation is the demographic makeup of our participants, which was predominantly older and white (S6 Fig in S1 File), and may not fully represent behaviors of other age and ethnic groups that contribute to tick encounters. For example, white or Caucasian Americans may be more likely to engage in outdoor recreational activities such as hiking or camping [71,72]. Although this pattern is common in community science studies [73,74], we attempted to reduce barriers to participation by mailing tick kits directly to participants who could not retrieve them. Finally, another limitation of our study was the inability to provide infection status of ticks to participants. Our rationale was to avoid influencing participants’ medical decision-making and the potential liability. However, this may have hindered participation, as participants frequently inquired about testing results via email or expressed dissatisfaction in returned surveys about not receiving individual results. Nonetheless, we are preparing an extension report for participating counties that summarizes aggregated county-level tick infection data, which may be shared with participants and other interested parties. Future studies using a similar model should consider providing individual results, if feasible, to improve participation. Despite these limitations, our results provide valuable insight into human behaviors and tick encounter patterns, and highlight opportunities for future research to broaden participation, improve data accuracy, and enhance public health recommendations to reduce TBD exposure.

Overall, our study provides a foundation for future community science efforts focused on tick-borne diseases. We emphasize the value of collaborations between researchers and local health and vector control agencies to strengthen research, surveillance, and public engagement as TBD threats continue to grow in the United States. Although not significant, our results also provide insight into human behaviors that may contribute to tick exposure in North Carolina. Future studies can build on this framework to enhance participation from local residents and health agencies, improve data collection, and ultimately develop actionable strategies to reduce tick-borne disease risk.

## Supporting information

S1 File A file containing S1-S9 Figures. (DOCX)

S2 File A file containing S1-S2 Tables. (DOCX)

S3 File Tick survey. Survey provided to participants to collect information on tick encounters. Completion was required for participation.
(PDF)

S4 File Provider survey. Survey provided to personnel at participating local health agencies to collect information on perceptions of project participation, as well as advertising type and frequency.
(PDF)
