# Supplementary material for: It takes a village: Community science informs tick encounter, pathogen, and exposure risk in North Carolina, USA
Source: PLoS One. 2026 Jul 24;21(7):e0352204. doi: 10.1371/journal.pone.0352204 (PMC13399343; doi:10.1371/journal.pone.0352204)
Supplement: S1 File — (DOCX) [file pone.0352204.s001.docx]

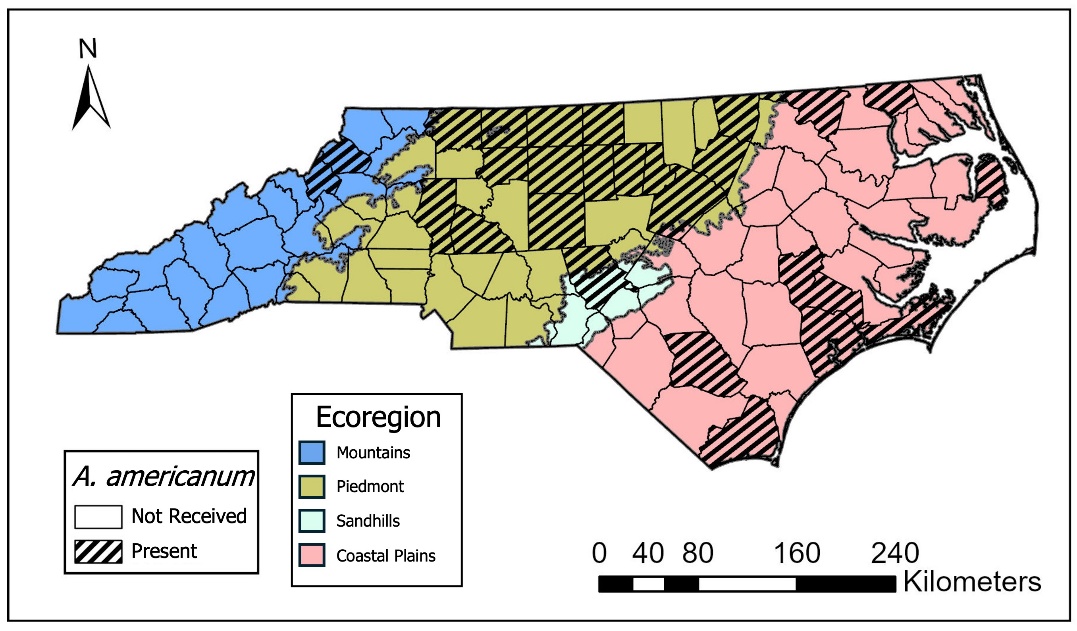


**S1 Fig.** Geographic distribution of *Amblyomma americanum* submissions, with counties shaded to indicate presence. Basemap: North Carolina Ecoregions.


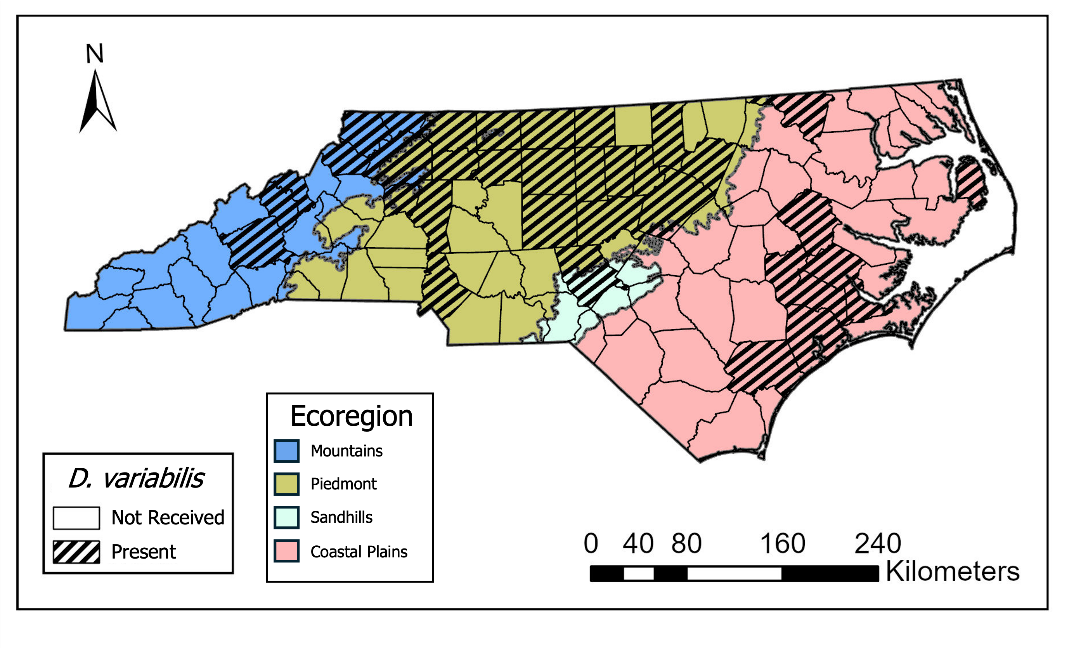


**S2 Fig.** Geographic distribution of *Dermacentor variabilis* submissions, with counties shaded to indicate presence. Basemap: North Carolina Ecoregions.


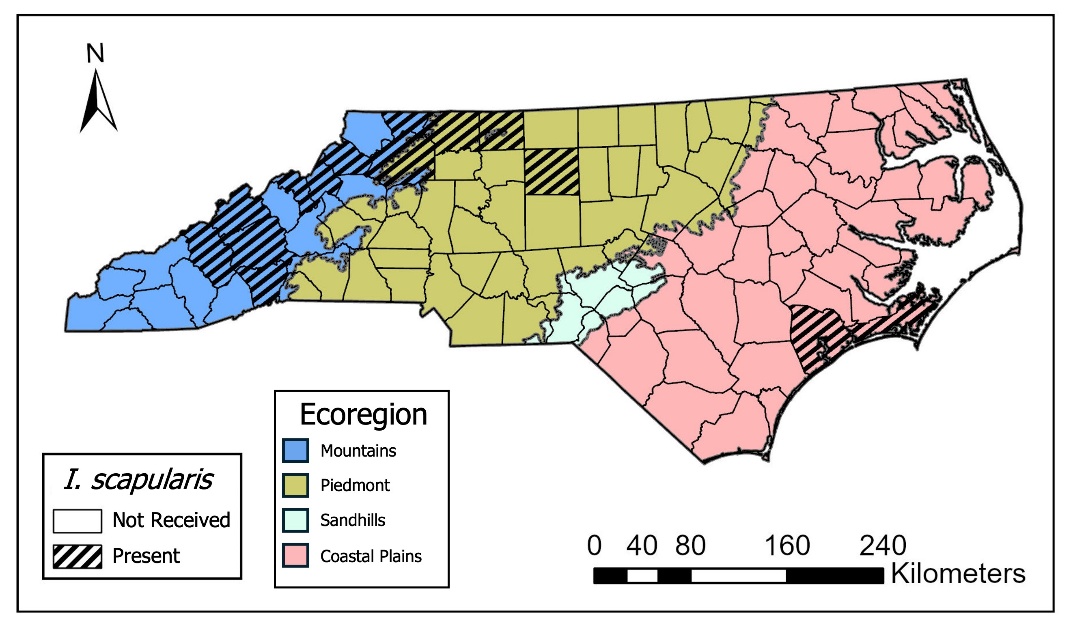


**S3 Fig.** Geographic distribution of *Ixodes scapularis* submissions, with counties shaded to indicate presence. Basemap: North Carolina Ecoregions.


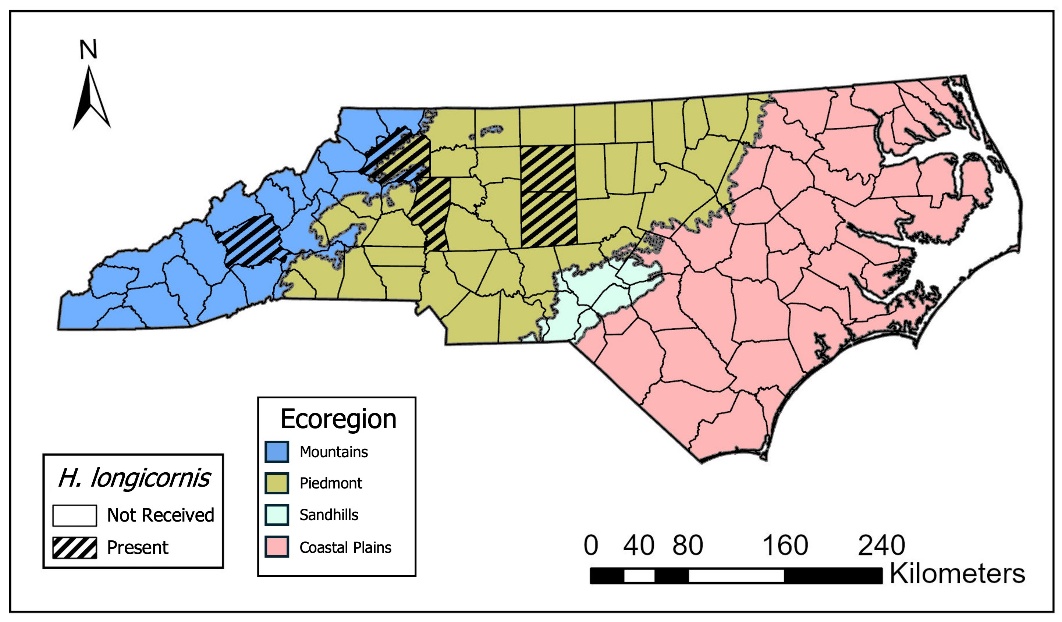


**S4 Fig.** Geographic distribution of *Haemaphysalis longicornis* submissions, with counties shaded to indicate presence. Basemap: North Carolina Ecoregions.


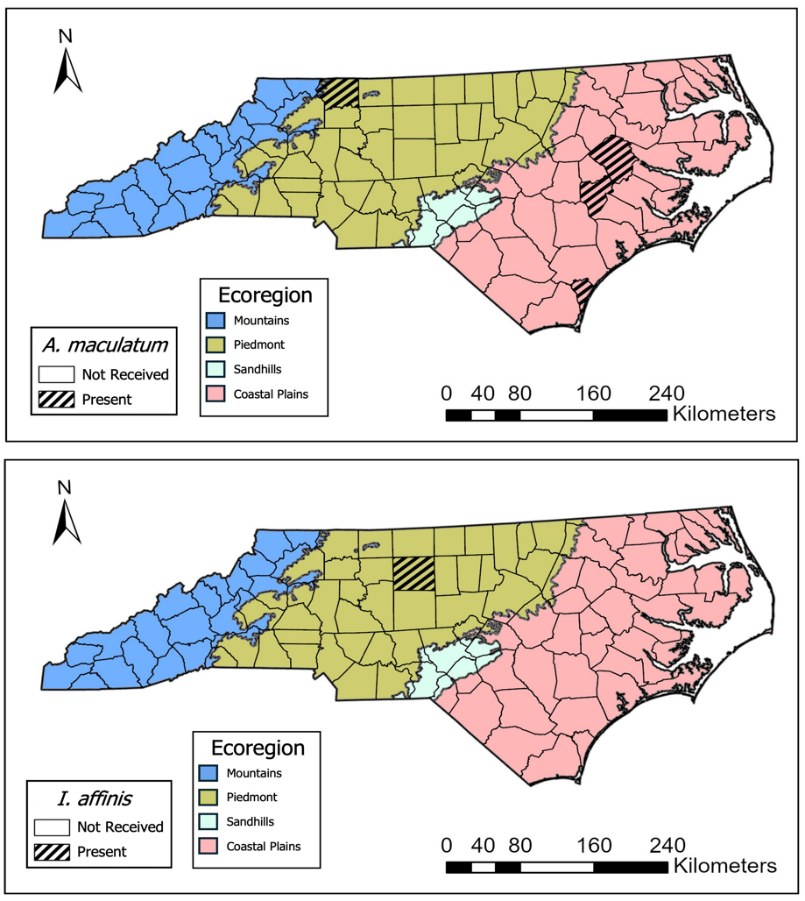


**S5 Fig.** Geographic distribution of *Amblyomma maculatum* (left) and *Ixodes affinis* (right) submissions, with counties shaded to indicate presence. Basemap: North Carolina Ecoregions.


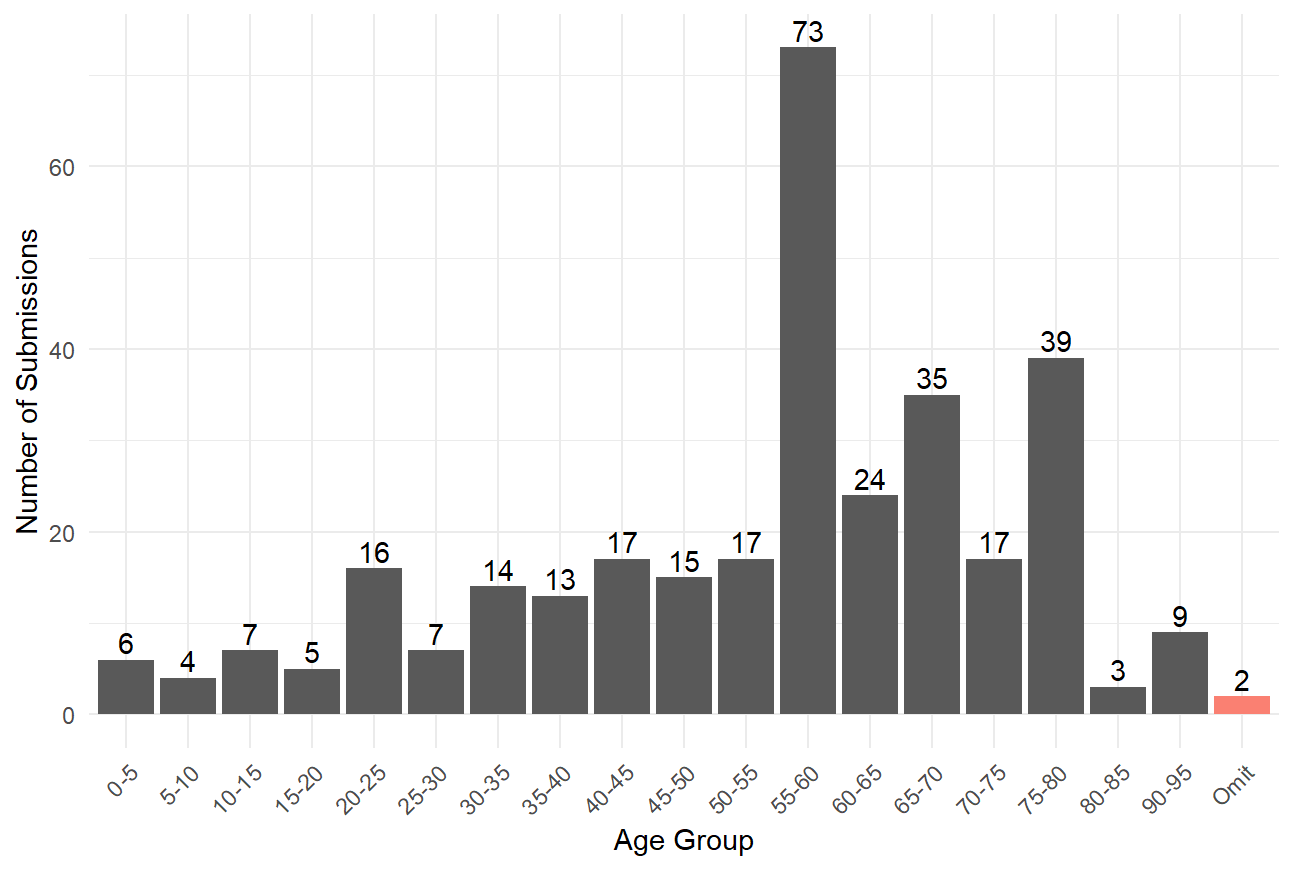


**S6 Fig.** Distribution of reported participant age groups for tick kit submissions. For submissions containing multiple ticks, a single age was recorded unless more than one age was reported on the survey form. Participant ages ranged from 3 to 90 years, with a median age of 58 years (IQR: 41–68). Submissions from participants who did not report an age are also included.


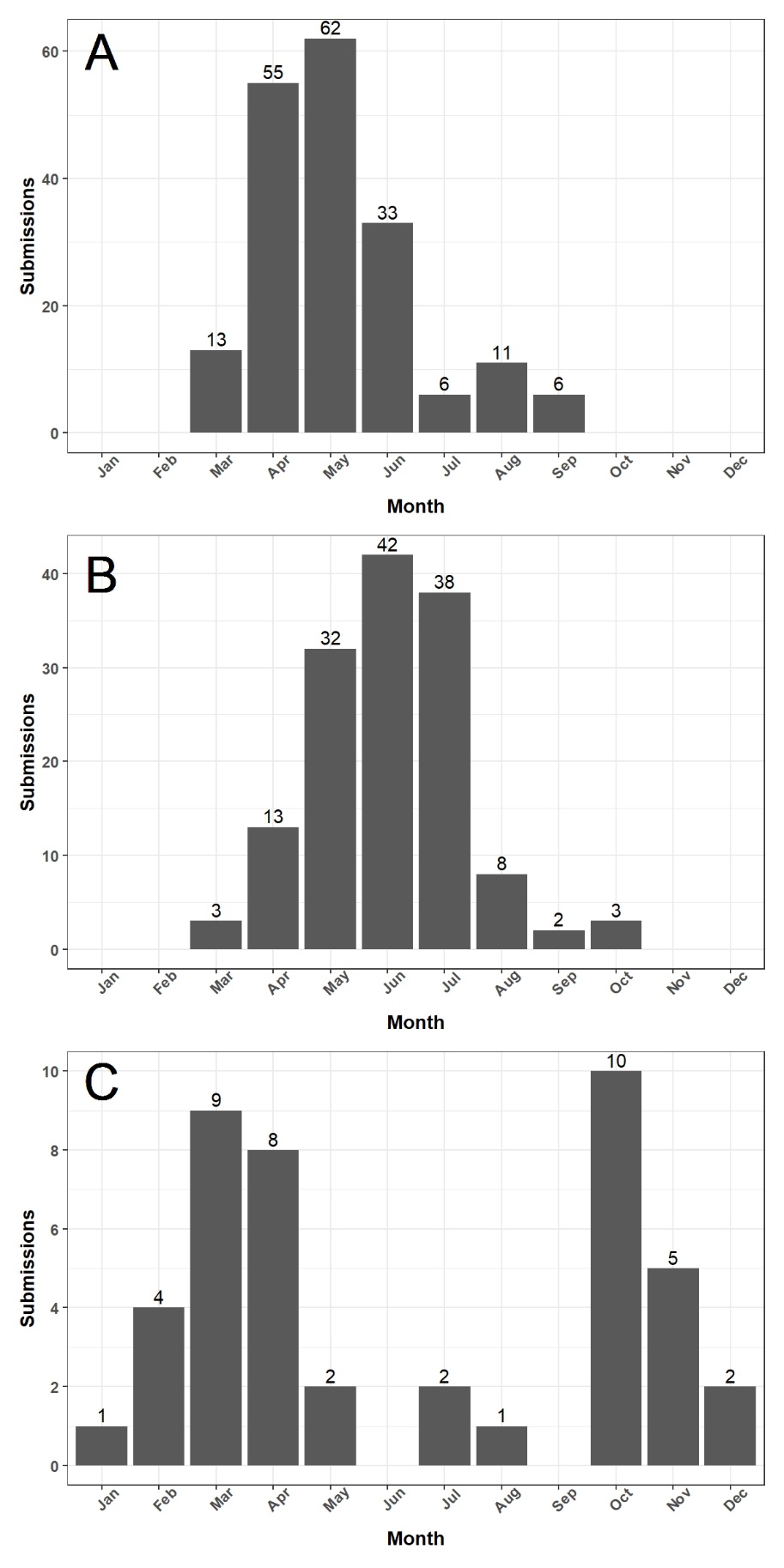


**S7 Fig.** Phenology of *Amblyomma americanum* (A), *Dermacentor variabilis* (B), and *Ixodes scapularis* (C) submissions over two years.


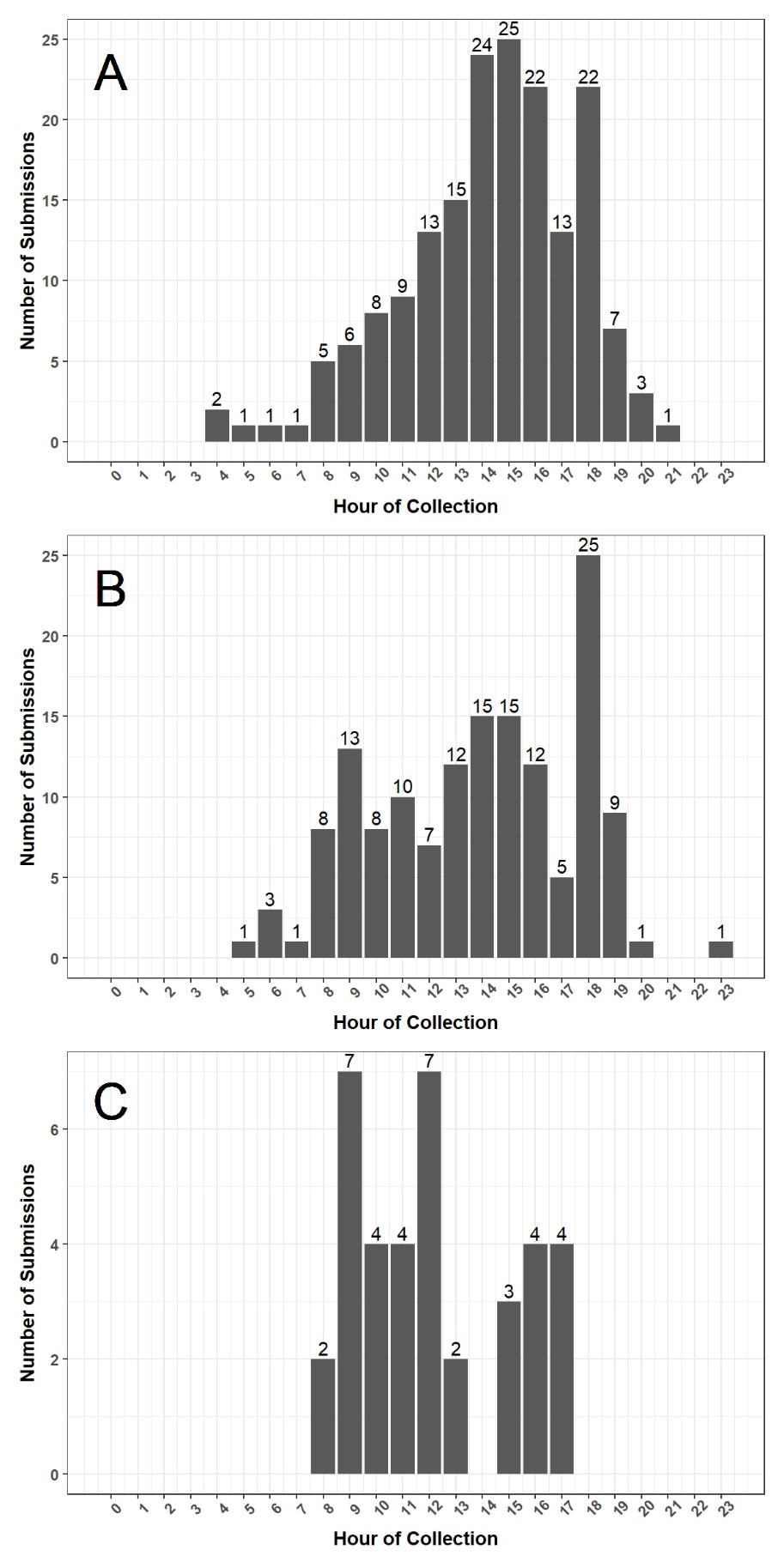


**S8 Fig.** Reported collection times for *Amblyomma americanum* (A), *Dermacentor variabilis* (B)*,* and *Ixodes scapularis* (C) ticks across the study period.


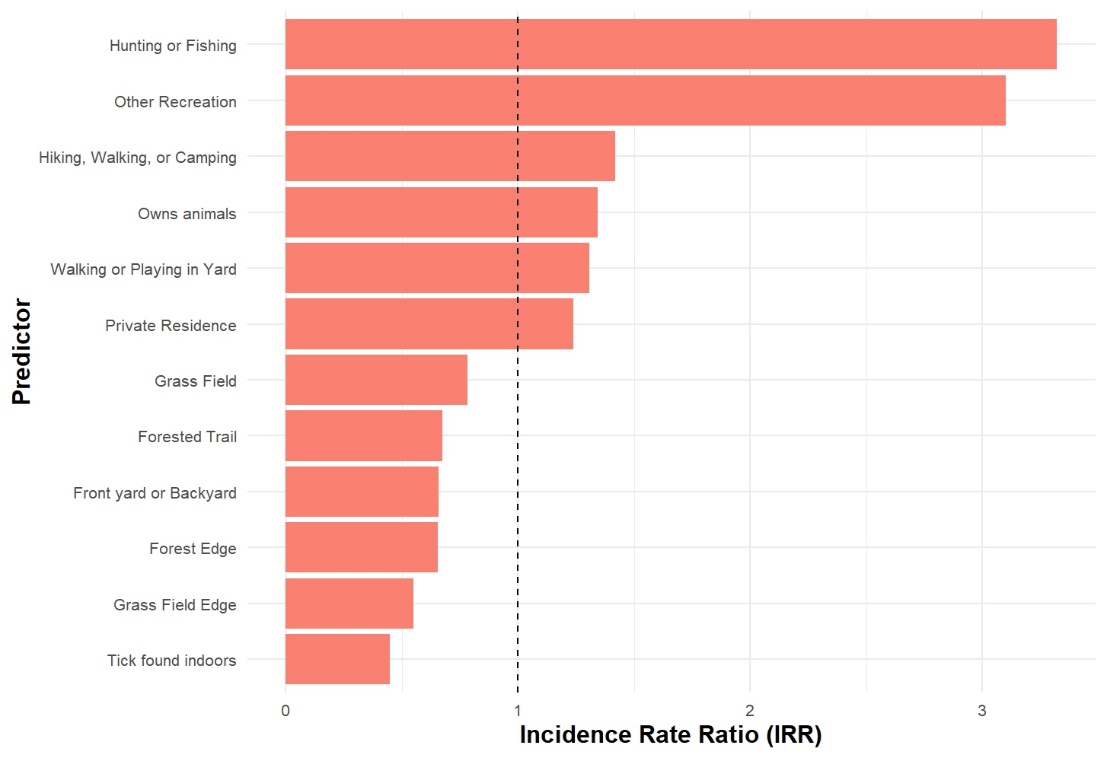

**S9 Fig.** Bar plot showing the incidence rate ratios (IRRs) of predictors in our best-fit model for tick kit submissions. Predictors are ordered by effect size, with the strongest predictors at the top. An IRR greater than 1 indicates a stronger association with tick kit submissions, while an IRR less than 1 indicates a weaker association.
