# Supplementary material for: It takes a village: Community science informs tick encounter, pathogen, and exposure risk in North Carolina, USA
Source: PLoS One. 2026 Jul 24;21(7):e0352204. doi: 10.1371/journal.pone.0352204 (PMC13399343; doi:10.1371/journal.pone.0352204)
Supplement: S2 File — (DOCX) [file pone.0352204.s002.docx]

| **Variable** | **Description** |
| --- | --- |
| Outdoor Recreation | Broad category coded when any recreational activity was mentioned |
| Outdoor Work | Broad category coded when any type of outdoor work was mentioned |
| Yard Work | Subset of outdoor work coded when participants described working in their yard |
| Private Residence | Coded when participants stated the tick was collected on their property |
| Pets/Animals | Coded when participants described the presence of pets or farm animals (e.g., dog walking) |
| Gardening | Subset of outdoor recreation and outdoor work coded when gardening was specifically mentioned |
| Hunting/Fishing | Subset of outdoor recreation coded when participants stated they were hunting or fishing. |
| Hiking/Walking/Camping | Subset of outdoor recreation coded when participants described hiking, walking, or camping activities |
| Walking or playing in residential yards | Coded when participants described walking or playing in residential yards |
| Other Recreational Activities | Coded when participants described less frequently reported recreational activities |
| Indoors | Coded when participants described finding the tick indoors without associated outdoor activity |
| Forested Trail | Coded when participants described walking or hiking on a recreational trail |
| Forest Edge | Coded when participants described being at the edge of a forest ecotone |
| Grass Field | Coded when participants described being in a grass field |
| Grass Field Edge | Coded when participants described being at the edge of a grass field ecotone |
| Residential Yard | Coded when participants described being at the edge of a grass field ecotone |
| Buildings | Coded when participants described being inside a man-made structure, including houses, barns, or sheds |
| Nearby Body of Water | Coded when participants described being near a lake, river, stream, or similar body of water. |

**S1 Table.** Explanatory variables recorded from surveys and considered in the full model prior to variable removal, including descriptions of how each variable was coded from survey responses. All variables were binary and coded as either Yes or No. In addition to the binary variables, a population offset variable was included to account for variation in population size among participating counties.

| **County** | **Kits Provided** | **Kits Received** |
| --- | --- | --- |
| Alamance | 5 | 3 |
| Alexander | 0 | 2 |
| Alleghany | 100 | 10 |
| Ashe | 100 | 1 |
| Avery | 50 | 3 |
| Bladen | 0 | 1 |
| Brunswick | 56 | 0 |
| Buncombe | 132 | 10 |
| Burke | 6 | 0 |
| Cabarrus | 0 | 1 |
| Camden | 5 | 0 |
| Carteret | 0 | 1 |
| Chatham | 0 | 5 |
| Craven | 0 | 1 |
| Currituck | 6 | 0 |
| Dare | 100 | 3 |
| Davidson | 11 | 1 |
| Duplin | 50 | 0 |
| Durham | 12 | 6 |
| Forsyth | 46 | 14 |
| Franklin | 6 | 4 |
| Gates | 4 | 0 |
| Guilford | 189 | 95 |
| Henderson | 0 | 1 |
| Hoke | 100 | 0 |
| Iredell | 150 | 7 |
| Jones | 50 | 2 |
| Lenoir | 100 | 3 |
| Macon | 5 | 0 |
| Madison | 0 | 1 |
| Mecklenburg | 10 | 1 |
| Mitchell | 150 | 8 |
| Moore | 12 | 6 |
| New Hannover | 112 | 1 |
| Northampton | 150 | 4 |
| Onslow | 100 | 5 |
| Orange | 11 | 6 |
| Pasquotank | 0 | 1 |
| Pender | 50 | 1 |
| Pitt | 100 | 5 |
| Randolph | 29 | 10 |
| Rockingham | 168 | 28 |
| Rowan | 0 | 2 |
| Stokes | 18 | 17 |
| Surry | 156 | 26 |
| Wake | 495 | 12 |
| Warren | 0 | 2 |
| Watauga | 150 | 6 |
| Yancey | 100 | 3 |

**S2 Table.** Number of kits provided to county health agencies and the number returned by participants within those counties. Some counties that were not provided with kits still returned kits to NCSU. Although 22 counties were formally enrolled in the study, interested participants could contact us directly to request mailed kits, which were included in the total number of kits provided.
