# Supplementary material for: It takes a village: Community science informs tick encounter, pathogen, and exposure risk in North Carolina, USA
Source: PLoS One. 2026 Jul 24;21(7):e0352204. doi: 10.1371/journal.pone.0352204 (PMC13399343; doi:10.1371/journal.pone.0352204)
Supplement: S3 File — Survey provided to participants to collect information on tick encounters. Completion was required for participation. (PDF) [file pone.0352204.s003.pdf]

<u>Survey Instructions</u>

Please fill out this survey as accurately as possible. No information will be shared, and you will not be identified from your responses. If you cannot remember the exact time and date of collection, please estimate approximately. If you are filling this survey out for a child, please fill in the appropriate survey information for the child.

1. What kind of tick do you think this is?

2. What were you doing when you picked up the tick(s)?

3. Where did you pick up the tick(s)?
   Please include at least a county of collection
   (ex: White Pines Nature Preserve, Chatham County)

4. At what approximate time and day did you pick up the tick(s)? (Not when you found the tick on you)

5. What type of habitat were you in when you picked up the tick? (ex: Forest, Tall grass, etc.)

6. County of Residence:
7. Ethnicity:
8. Age:
9. Gender:
10. If you would like general information about the tick(s) you are submitting including identification and prevention, please provide an email address, and NC State Entomology will respond as soon as possible.
    Note: NCSU can't provide testing information for your submitted ticks.
