# Supplementary material for: It takes a village: Community science informs tick encounter, pathogen, and exposure risk in North Carolina, USA
Source: PLoS One. 2026 Jul 24;21(7):e0352204. doi: 10.1371/journal.pone.0352204 (PMC13399343; doi:10.1371/journal.pone.0352204)
Supplement: S4 File — Survey provided to personnel at participating local health agencies to collect information on perceptions of project participation, as well as advertising type and frequency. (PDF) [file pone.0352204.s004.pdf]

Tick Kit Supplier Survey Questions

1. What was your role in distributing kits to participants?

2. In which county were the tick kits distributed?

3. How did your county first hear about the citizen science project?

4. Did your county recommend another county to distribute tick kits? If yes, please specify which county.

5. Where in your department was the kiosk located?

6. What types of advertising did your department use to inform residents about the kiosks and the citizen science program?

7. How often did your department advertise the citizen science program?

   (0) Never
   (1) Once
   (2) Twice
   (3) Three to five times
   (4) Greater than five times

8. Did your county experience any challenges related to kit distribution? If so, please describe any experienced challenges.

9. Why did your county health department enroll in this citizen/community science project? Select all that apply.

    a. Concern about tick-borne diseases spreading into our community
    b. Concern about tick-borne diseases already in our community
    c. To contribute to scientific research efforts in our community
    d. Public awareness of tick-borne diseases
    e. Interest in participating in a citizen/community science project
    f. Other (please describe below)

10. Would your county participate in another citizen/community science project hosted by NCSU or another university again in the future? If not, please specify why.

11. Do you have any additional comments or suggestions about the project?

12. Please rank the following methods for receiving project results, with '1' being your top choice and '4' being your least preferred.

Peer-reviewed publication	_____
Extension newsletter	_____
Email update	_____
Public event/community outreach event	_____

13. Please indicate how concerned you are about tick-borne diseases in your county.

(5) Not at all concerned
(6) Slightly concerned
(7) Moderately concerned
(8) Concerned
(9) Very concerned

14. How often does your county receive reports or hear concerns of tick-related illnesses?

(0) Never
(1) Once a year
(2) Once a month
(3) Once a week
(4) Almost daily

15. How interested do you think the community was in participating in the citizen science project?

(0) Not at all interested
(1) Slightly interested
(2) Moderately interested
(3) Interested
(4) Very Interested

16. How effective do you think the tick kit kiosks were at promoting engagement in the citizen/community science project?

(0) Not at all effective
(1) Slightly effective
(2) Moderately effective
(3) Effective
(4) Very effective
